# Broadband multiresonator quantum memory-interface

**DOI:** 10.1038/s41598-018-21941-6

**Published:** 2018-03-05

**Authors:** S. A. Moiseev, K. I. Gerasimov, R. R. Latypov, N. S. Perminov, K. V. Petrovnin, O. N. Sherstyukov

**Affiliations:** 1 Kazan Quantum Center, Kazan National Research Technical University n.a. A.N.Tupolev-KAI, 10 K. Marx, Kazan, 420111 Russia; 2 Kazan Federal University, 18 Kremlyovskaya Str., Kazan, 420008 Russia

## Abstract

In this paper we experimentally demonstrated a broadband scheme of the multiresonator quantum memory-interface. The microwave photonic scheme consists of the system of mini-resonators strongly interacting with a common broadband resonator coupled with the external waveguide. We have implemented the impedance matched quantum storage in this scheme via controllable tuning of the mini-resonator frequencies and coupling of the common resonator with the external waveguide. Proof-of-principal experiment has been demonstrated for broadband microwave pulses when the quantum efficiency of 16.3% was achieved at room temperature. By using the obtained experimental spectroscopic data, the dynamics of the signal retrieval has been simulated and promising results were found for high-Q mini-resonators in microwave and optical frequency ranges. The results pave the way for the experimental implementation of broadband quantum memory-interface with quite high efficiency *η* > 0.99 on the basis of modern technologies, including optical quantum memory at room temperature.

## Introduction

The development of the quantum memory (QM) as well as its effective light-media quantum interface are of decisive importance for quantum information technologies^1–3^. Impressive experimental results on the way to the effective optical QM were achieved in the last decade^4–6^. Recently the developed approaches stimulated active studies for the elaboration of the microwave QM which becomes a key element for the creation of multiqubit superconducting quantum computer^7–10^. QM should be able to store many short pulses with the high efficiency^11^ and to satisfy very strong requirements of multiqubit quantum processing and error correction procedures^12^. In the practical implementation of long-lived multiqubit QM, it is assumed to satisfy a sufficiently strong and reversible interaction of light/microwave qubits with many^13,14^ information carriers, in particular with NV-centers in diamond^15^ and rare-earth ions in inorganic crystals^16^. Implementation of sufficiently high quantum efficiently remains main problem in elaboration of the QMs.

One of the promising approaches to constructing a QM is based on the spin/photon echo effect in resonant ensembles of atoms and electron spins^17–20^, where a strong coupling of light/microwave photons with quantum electrodynamics cavity mode also plays a crucial role for the effective reversible transfer of quantum information from the flying qubits to the long-lived atomic/spin coherence^7,21–26^. It is possible to increase the quantum efficiency for the broadband interface in this approach via spreading the impedance matching condition to a wider range of working frequencies^22^. However a satisfactory solution of this problem remains unknown for high-Q resonator that strongly limits the spectral width of QM by the overly narrow resonator linewidth^9^.

In this work, starting from the AFC (Atomic Frequency Comb) protocol of the photon echo QM^27^ in the single mode cavity^21^, we showed that quantum memory-interface (QMI) can be efficiently implemented for broadband electromagnetic pulses on a system of high-Q mini-resonators. Herein, we focus on experimental demonstration of this approach in microwave spectral range. To enhance dramatically the interaction with the signal field, we used a set of a small number of mini-resonators coupled to a common broadband resonator that makes it possible to achieve a quite strong interaction of pulse with the mini-resonators which provided highest quantum efficiency of 16.3 % for the broadband storage of microwave pulses. On the basis of the experimental data obtained in the network analyzer measurement, we restored the internal parameters and simulated the observed dynamics of the fabricated multiresonator (MR) scheme with high agreement.

The constructed QMI setup has demonstrated promising technical properties: compactness, low cost and ease of fabrication, which are convenient for controlling the field dynamics and integration of the MR scheme into the microwave circuits of quantum processing. This technical solution allowed us to experimentally investigate the basic fundamental properties of the MR scheme, and we have shown that such QMI can be implemented on different on-chip platforms for optical and for microwave spectral range^28–31^ with the considerably higher quantum efficiency *η* > 0.999 at sufficiently low temperatures.

## QMI Setup

**Figure 1:**
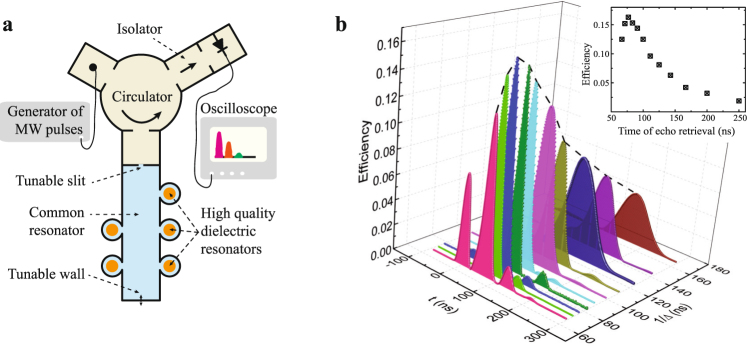
(**a**) Experimental installation of the MR QMI scheme (blue color of filling): the large common resonator is connected with 5 cylindrical brass mini-resonators, the frequencies of which can be tuned by using the handles fixing the lengths of the cylinders. (**b**) Reflected (t = 0) and echo signals from MR circuit at the varied spectral detuning Δ between mini-resonators. Dashed envelope and black square dots in the insert of figure (**b**) show the dependence of the efficiency on the storage time. Size of the points in the inset corresponds to experimental error. Signals were normalized to the amplitude of input signals, which are not shown.

The QMI setup and full experimental installation are shown in Fig. 1a. The setup contained five cylindrical brass resonators (mini-resonators) filled by the dielectric inserts (small cylindrical resonators) characterized by the quite large dielectric permittivity *ε* ~ 29. The mini-resonators had a variable length to provide fine tuning of the resonant frequency. Each mini-resonator was coupled to the large common rectangle broadband resonator through a thin nontunable slit. The large resonator was made of a copper waveguide (23 × 10 mm). It had one tunable wall to adjust its resonator frequency to provide a strong coupling of the input field with the mini-resonator system. The input tunable slit to the common resonator can be partially or fully open. The last regime was used to adjust the frequencies of mini-resonators to the periodic spectral comb with equal spectral distances Δ (see an example of the experimental spectrum in the section Physical model). An Agilent PNA-X microwave network analyzer was used in spectral measurements and tuning the mini-resonator frequencies. For this purpose in section Physical model we also presented numerical studies of the experimental spectroscopic parameters and used these parameters in the theoretical simulation of echo experiments.

## Experiment

**Figure 2:**
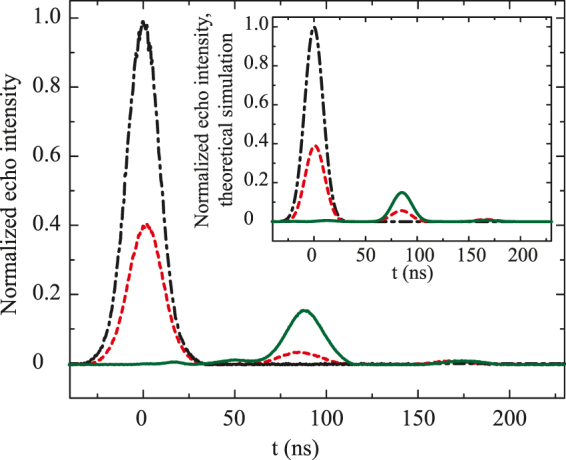
Experimental curves of the normalized echo intensity for Δ = 12 MHz: black dot-dashed line–input pulse, red dashed line–echo for open configuration (when the input slit is fully open), green solid line–echo for optimal *κ* (impedance matching condition). Theoretical curves of the normalized echo intensity for Δ = 12 MHz are shown in the insert. Type and color of the lines for echo at the optimal *κ* are the same as in the experimental curves in Fig. 1b.

An Elexsys E580 ESR spectrometer (Bruker) at X-band (9.8 GHz) was used in the preliminary echo experiments, where the efficiency of the retrieved echo was ~4% that corresponds to the theoretical red (dashed) curve in Fig. 2. In these experiments, only broadband microwave pulses with the rectangular form can be used. Such pulses had no possibility for the effective implementation of the impedance matching condition and led to the very limited efficiency.

To improve the impedance matching condition, we used a home-built setup (Fig. 1a). An Agilent E8267D vector signal generator with the amplitude modulation by an arbitrary waveform generator was used here as a source of microwave pulses with the Gaussian temporal form. The generator was coupled to the rectangular waveguide and through a standard X-band circulator to MR QMI. The reflected signal and emitted echo pulses were detected by the detector section. We used an isolator to avoid small undesirable signals reflected from the detector section and passed again to the generator. In our experiment, the power of pulses could be varied from −18 to +18 dBm. We performed experiments to observe the echo at different power of the input signal, started from 0 dBm. The observed efficiency did not depend on the power of input signal and following echo-experiments were performed at power of 10 dBm. We have performed a series of experiments with different spectral detunings Δ varied between 4 and 15 MHz as shown in Fig. 1b. Microwave classical Gaussian pulses with the spectral width up to 48 MHz were stored in MR QMI and then were retrieved automatically by MR QMI in the time range of 66–250 ns. As expected, the time of the echo emission was 1/Δ and the efficiency strongly depended on the detuning Δ as shown in the insert of Fig. 1b. The size of the points of this dependence corresponds to an experimental measurement error, which was estimated from series of experiments with small variations of the parameters of the QMI (frequency tuning of mini-resonators, tuning of the coupling and frequency of the large common resonator). The dependence of the echo efficiency on the detuning Δ is in qualitative agreement with the analytical estimation of the quantum efficiency *η* (1).

We found the analytical estimation of the first echo pulse retrieval by using Equation (2) (see section Physical model) at the optimal coupling *κ*:

1 $$\eta ={(1+\frac{2{\gamma }_{r}{\rm{\Delta }}}{{g}^{2}})}^{-2}\exp \{-2\gamma /{\rm{\Delta }}\},$$

where we assumed the pulse spectrum $\delta {\omega }_{f}\pi \sim (N-1)\pi {\rm{\Delta }}$, *g* = 〈*g*_*n*_〉 and *γ* = 〈*γ*_*n*_〉 are the average coupling constant and decay rate of mini-resonators, respectively, Δ = 〈Δ_*n*+1_ − Δ_*n*_〉 is the spectral distance between mini-resonators, *T*_1_ = 1/Δ is the time of the signal recovery, the optimal coupling *κ* = *κ*_0_ = 2*γ*_*r*_ + *g*^2^/Δ of the common resonator with the external waveguide is determined by the impedance matching condition. For arbitrary *κ* the estimation for efficiency is given by the formula *η* = *g*^4^/(Δ^2^*κ*^2^)exp{−2*γ T*_1_}.

The maximal achieved efficiency for the echo signal retrieval was 16.3 % with the time delay ~77 ns (Δ = 13 MHz). In this case the reflection of the input signal is highly suppressed due to the efficient impedance matching condition. One can see some partial reflection of the input pulse for other detunings Δ in Fig. 1. We explain this fact by the complexity of the experimental setup leading to limited experimental capabilities for the perfect control of the impedance matching at different parameters of MR system. The reflection coefficient at the central frequency *ω* = 0 of MR system *R* = [*κ* − 2*γ*_*r*_ − *g*^2^/Δ]^2^/[*κ* + 2*γ*_*r*_ + *g*^2^/Δ]^2^.

The results of echo experiments for detuning Δ = 12 MHz are also shown in Fig. 2, where the black (dot-dashed) curve is the input microwave pulse. The red (dashed) curve describes the reflected and two echo pulses when five coupled mini-resonators are connected to the external waveguide (the adjustable input slit of the large resonator was fully open). The green (solid) curve corresponds to the case when mini-resonators were included in the tunable large common resonator. Herein, the large resonator was coupled to the external waveguide where the coupling constant *κ* was tuned to achieve the impedance matching condition. In this case, the efficiency of the echo emission also highly increased up to 15.4 %, while the second echo was almost absent as it is seen from the comparison of red (dashed) and green (solid) curves of Fig. 2 and the reflected signal at *t* = 0 was also almost absent. As it is seen in Fig. 2, the temporal shape of echo pulse coincides well with the shape of input pulse that indicates to the quite good fidelity of the studied storage.

Numerical simulations of experimental data performed by using Equation (3) and spectroscopic parameters of the MR system (see section Physical model) are presented in the insert of Fig. 2. The comparison of experimental and theoretical curves demonstrates rather good agreement of these data and shows that the quantum efficiency of the MR scheme is mainly determined by the decay constants *γ*_*r*_ and *γ*_*n*_, i.e., by the ohmic losses in the common broadband resonator and in the system of mini-resonators.

## Physical Model

**Figure 3:**
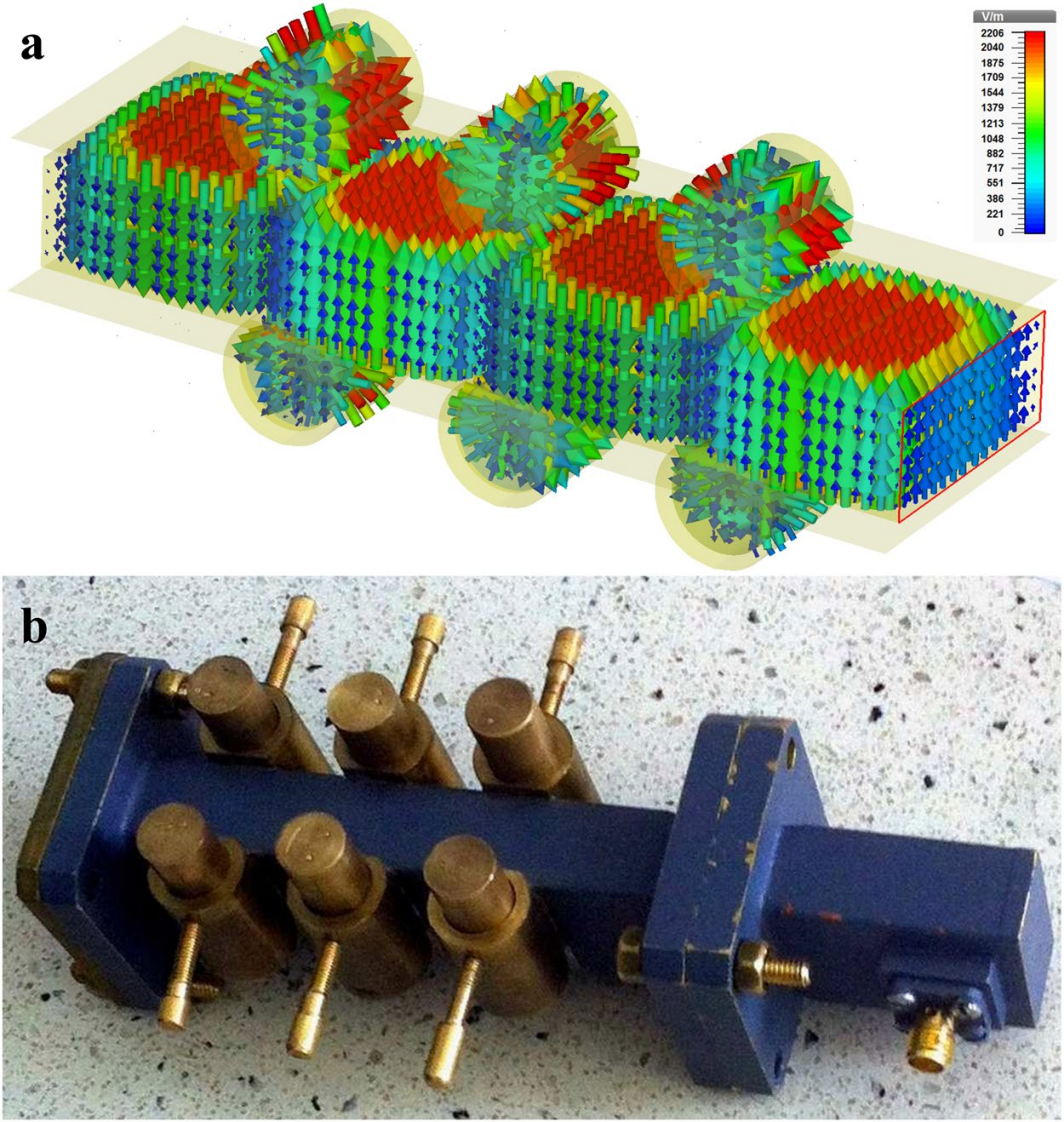
(**a**) Simulation of the microwave field in the QMI system using the “CST Studio Suite 2015” program. The color arrows show the amplitude of the electric field strength in the resonators. (**b**) Fabricated setup used in our experiments.

We simulated a QMI setup based on the MR system^32^ connected with the common broadband resonator. The “CST Studio Suite 2015” program was used for the optimization of geometric parameters of the mini-resonators and their spatial positions. Operating modes of the electromagnetic field were TM_104_ and TE_010_ for the large resonator and mini-resonators, respectively. The coupling of mini-resonators to the large common resonator was implemented through the magnetic field by using rectangular slits. The parameters of these slits were also optimized by using the “CST Studio Suite 2015” program. The results of the QMI system simulation and the fabricated setup are shown in Fig. 3. This simulation allowed us to estimate the optimal geometric parameters of the setup (the size of the slits, mini-resonators and their locations) for manufacturing the experimental model. We also note that in the echo experiment we used only 5 mini-resonators of 6 to simplify the adjustment of the parameters and to increase the range of the Δ value.

The theoretical model of the considered memory corresponds to the so-called impedance matching QM on the photon echo in a single mode cavity^21,22,33^, which was expanded further to the system of ring resonators connected with the nanooptical fiber^34^ and to other integrated schemes^32,35^. Using the input-output formalism of quantum optics^36^ for the system studied in the work, we obtain the equations for field modes of mini-resonators *s*_*n*_(*t*) and of the common cavity field *a*(*t*):

2 $$\begin{array}{c}{[{\partial }_{t}+i2\pi {{\rm{\Delta }}}_{n}+{\gamma }_{n}]}{s}_{n}(t)+{g}_{n}^{\ast }a(t)+{f}_{n}(t)= 0,\\{[{\partial }_{t}+{\frac{\kappa }{2}}+i2\pi {{\rm{\Delta }}}_{r}+{\gamma }_{r}]}a(t)+{f}_{r}(t)-\sum _{n}{g}_{n}{s}_{n}(t)= {\sqrt{\kappa }}{a}_{in}(t),\end{array}$$

where *a*_*in*_(*t*) = [2*π*]^−1/2^∫ *dωe*^−*iωt*^*f*_*ω*_ is the input pulse, *f*_*ω*_ is the spectral profile of the input pulse, for which the normalization condition is fulfilled ∫*dω*|*f*_*ω*_|^2^ = 1 for the single photon field, *ω* is the circular frequency counted from the central circular frequency of the radiation *ω*_0_, *ν* = *ω*/(2*π*) is the conventional frequency, Δ_*n*_ are frequency detuning of mini-resonators, *n* ∈ {1, ..., *N*}, *γ*_*n*_ is the decay constant of the field mode in *n*-th mini-resonator, *κ* is the coupling coefficient of the broadband resonator with the external waveguide, *g*_*n*_ is the coupling constant of the common resonator mode with the field mode of the *n*-th mini-resonator, Δ_*r*_ is the frequency shift of the broadband resonator mode and *γ*_*r*_ is the decay constant of this mode; *f*_*n*_(*t*), *f*_*r*_(*t*) are the delta-correlated Langevin forces satisfying the following relations $< {f}_{n,r}^{\dagger }(t) > = < {f}_{n,r}(t) > =0$ and $< {f}_{n,r}^{\dagger }(t){f}_{n,r}(t^{\prime} ) > ={\gamma }_{n,r}\delta (t-t^{\prime} )$^37^.

Below we focus only on the spectral quantum efficiency of the signal retrieval which is found from (2) by ignoring the Langevin forces (see, for example^38^). From Equation (2) we obtain the output field *a*_*out*_(*t*) = (*κ*)^1/2^*a*(*t*) − *a*_*in*_(*t*) in terms of the transfer function $S(\omega )={\tilde{a}}_{out}(\omega )/{\tilde{a}}_{in}(\omega )$ in the form

3 $$\begin{array}{rcl}S({\omega }) & = & \frac{1-F({\omega })}{1+F({\omega })},\\ F({\omega }) & = & \frac{2({\gamma }_{r}+i2\pi {{\rm{\Delta }}}_{r}-i{\omega })}{\kappa }+\sum _{n}\frac{2{g}_{n}^{2}}{\kappa ({\gamma }_{n}+i2\pi {{\rm{\Delta }}}_{n}-i{\omega })},\end{array}$$

where ${a}_{in,out}(t)={\mathrm{[2}\pi ]}^{-\mathrm{1/2}}\int d\omega {e}^{-i\omega t}{\tilde{a}}_{in,out}(\omega )$. In the general case transfer function (3) has a very complicated spectral behaviour due to the strong interaction of the mini-resonators in the common cavity^13,14,39^ and the exact recovery of the dynamics is difficult. The analytical estimation (1) can be obtained from the first Equation (2) by integrating and discarding the energy losses at the stage of fast loading the input pulse. Here we take into account that *a*_*in*_ = 0 while ${s}_{n}(\delta {t}_{s}\le t\le \mathrm{1/}{\rm{\Delta }})\cong -g{e}^{-(\gamma +i2\pi {{\rm{\Delta }}}_{n})t}\int d\tau {e}^{i2\pi {{\rm{\Delta }}}_{n}\tau }a(\tau )$ (where $\delta {t}_{s}\sim 2\pi /\delta {\omega }_{f} < \mathrm{1/}{\rm{\Delta }}$, *δω*_*f*_ ≅ 2*πN*Δ), Δ_*n*_ = (*n* − (*N* − 2))Δ is the absorption time of the signal field. The expression ${S}_{in}(t)={\sum }_{n}{g}_{n}{s}_{n}$ plays the role of external source in Equation (2) for the stage of the echo pulse emission: ${S}_{in}(t)=-\sqrt{2\pi }{g}^{2}{e}^{-\gamma t}{\sum }_{n}{e}^{-i2\pi {\Delta }_{n}t}\tilde{a}(2\pi {{\rm{\Delta }}}_{n})$. By taking into account that echo pulse is irradiated at *t* = 1/Δ in the factor *e*^−*γt*^ ≅ *e*^−*γ*/Δ^ and that Δ_*n*_ = (*n* − (*N* − 2))Δ, we pass from the sum ∑_*n*_ to the integral ${\sum }_{n}{e}^{-i2\pi {{\rm{\Delta }}}_{n}t}\tilde{a}(2\pi {{\rm{\Delta }}}_{n})\to \frac{1}{2\pi {\rm{\Delta }}}{\int }_{-N/2}^{N/2}dx{e}^{-ixt}\tilde{a}(x)=\frac{1}{2\pi {\rm{\Delta }}}{\int }_{-\infty }^{\infty }dx{e}^{-ixt}\tilde{a}(x)=\frac{\sqrt{2\pi }}{2\pi {\rm{\Delta }}}a(t)$. Therefore we get for the source of echo field ${S}_{in}(t)=\frac{-{g}^{2}}{{\rm{\Delta }}}{e}^{-\gamma /{\rm{\Delta }}}a(t)$. Substituting *s*_*n*_ in Equation (2) and taking the into account the estimation of *S*_*in*_(*t*), we evaluate the echo field intensity and efficiency (1), respectively. Note that the above approximate estimation of the optimal parameters and efficiency can be improved on the basis of the spectral-topological matching condition^40^.

**Figure 4:**
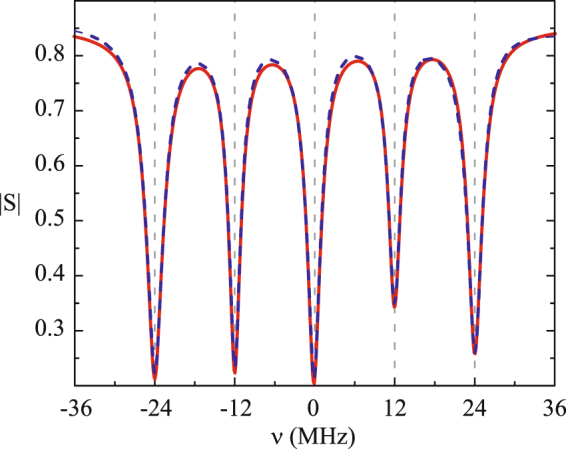
Spectrum |*S*(*ν*)| of the microwave QMI for Δ = 12 MHz: red solid line–theory, blue dashed line–experiment.

We measured the spectral characteristics of the QMI setup (5 mini-resonators that were used in the echo experiments) on an Agilent PNA-X network analyzer. Based on spectroscopic experimental data (see Fig. 4) obtained for Δ = 12 MHz and formula (3), which is true for single-photon and classical fields, we found the internal parameters of the MR system (further all the quantities are in units of MHz):

4 $$\begin{array}{rcl}{\omega }_{0} & = & 2\pi \cdot 9770,\,k=2\pi \cdot 293,\,{\gamma }_{r}=75,\,{{\rm{\Delta }}}_{r}=-3,\\ \{{g}_{n}\} & = & \{68,54,60,48,58\},\,g=58,\\ \{{\gamma }_{n}\} & = & \{8.6,6.2,6.8,6.8,7.4\},\,\gamma =7.3,\\ \{{{\rm{\Delta }}}_{n}\} & = & \{-24,-12,0,12,24\},\,{\rm{\Delta }}=12.\end{array}$$

The experimental and theoretical curves shown in Fig. 4 coincide with the high accuracy and we used all set of the obtained parameters of the MR scheme in section Echo and efficiency. The elaborated methods of the spectroscopic characterization of the MR scheme can be also applied for the simulation of fast switching of the mini-resonator couplings and frequencies^41–43^ which is required for the longer storage and on-demand retrieval of the absorbed microwave fields. These methods can work with switching time on the order of 100 ns that is applicable in our scheme. In addition, the QM devices in the microwave spectrum region^28^ assumes a more perfect experimental control of MR QMI parameters at fairly low temperature (around 10^−2^ K) that is necessary for protecting from thermal microwave quantum noises.

## Summary and Outlook

Thus we have implemented a proof-of-principal experiment for the storage of broadband microwave signals in the system of mini-resonators. Focusing on the obtained experimental result, we note that implemented MR system made possible the independent experimental control of the optimal spectral detuning Δ_*n*_, coupling constants *g*_*n*_ of mini-resonators and coupling constant *κ* with the external waveguide that provided the impedance matching condition for the storage of microwave pulses as it is seen in Fig. 2. Experimental results obtained under these conditions exhibit the quantum efficiency of 16.3 % which is the highest for the storage of broadband microwave pulses. Namely, the spectrum width of the input pulse is much larger compared to the linewidth of each mini-resonator field mode. It is worth noting that at room temperature similar quantum efficiency for broadband quantum storage (~15–27%) has been demonstrated for optical spectral range in atomic vapour^44,45^.

As it is seen in Fig. 2, the pulse storage is provided by the complete field silence in the common waveguide within some time interval after the absorption of the input pulse, before the first echo signal is emitted. During this time period, all input field energy is redistributed in the system of mini-resonators and is split into a number of uncoupled spectral blocks. At this stage one can disable the mini-resonators of the total resonator and to keep the input microwave field in them for a longer period of time and retrieve it later on demand. Moreover, since the mini-resonators are characterized by the higher Q-factor, it will be much easier to implement an interaction between the captured microwave field and the ensemble of electron spins located in the mini-resonators and therefore to provide the longer storage^16^ and even to process it independently in each high-Q mini-resonator. Thus, the proposed MR scheme opens new opportunities in comparison with well-known approaches to the implementation of efficient QM. The scheme provides the efficient storage of broadband microwave field in high-Q resonators. In comparison with AFC protocol in a single QED-cavity^21–23^, the proposed scheme can provide stronger interaction with long-lived spin systems due to the higher Q-factor of the mini-resonators. At the same time, MR scheme also demonstrated the possibility of broadband storage in comparison with using a single high-Q resonator^9^.

The possibility of using a small number of mini-resonators (microwave or optical) for efficient transfer of the broadband pulse from the common resonator to the spatially redistributed mini-resonators is a non-trivial problem. The problem is associated with the increase in the storage time of the signal and the implementation of the optimal topology for the connection of all the mini-resonators in a spatially small common waveguide. The problem is caused by the fact that the field modes of the mini-resonators begin to strongly interact with each other in the common resonator and change strongly their initial frequencies. These changes should be accurately reflected in the initial frequencies to provide final frequencies be periodic as it is required^27^ for the perfect QM. The super-high control of optimal spectral properties provides a superefficient quantum storage that is possible on the basis of the spectral-topological approach to MR QM^32,40^.

From the technological point of view, the proposed QMI scheme also indicates the possibility of its accurate implementation by the existing experimental methods^28,41,43^, by employing different physical platforms. For example one can use planar microwave waveguides characterized by highly reduced spatial sizes, various nanooptical waveguide structures, photonic crystals and other systems which make it possible to have localized interactions of many quantum point-like objects with a broadband carrier of signals. In particular, it seems especially useful to apply the nanooptical waveguides integrated with microcavities and LCFBGs^30,46^. In turn, the fast frequency switching of the optical microcavities can be operated by electro-optics and free-carrier dispersion control^47^. In the QMI scheme it is possible the internal parameters can be controlled fast^41,43^ together with the non-destructive control^48,49^, which makes it possible to use it as a high-efficiency broadband pre-processor for a quantum computer.

Coming back to the presented experiments, we note that the obtained result can be significantly improved at higher Q-factor of mini-resonators and/or at lower temperatures where decoherent processes will be highly suppressed. At helium temperature, the decay constants *γ*_*n*_ and *γ*_*r*_ could be much smaller in comparison with the coupling constants *g*_*n*_ and spectral detuning Δ^50^. In this case, the realistic estimation of quantum efficiency (1) of our system at such temperature gives *η* ≅ 1 − 4*γ*_*r*_Δ/*g*^2^ − 2*γ*/Δ ≅ 0.999 for $\gamma \sim {\gamma }_{r}\sim {10}^{-3}$ MHz and Δ ~ g ~ 4 MHz for microwave frequencies range. Similarly, by coupling a system of high-Q microresonators with common fiber optical resonator^34,51^, we find that efficiency of quantum storage can achieve *η* ≅ 0.99 for optical frequency range at room temperature. The room temperature for broadband optical quantum storage^44,45^ becomes possible due to the unique combination of the properties of QMI scheme when the system of high-Q microcavities can operate with broadband light pulses while keeping long-lived storage of light signal in each microresonator. These observations of microwave and optical microresonator QMI open the way for the construction a highly efficient broadband QMs by using current technologies^30,31,52,53^ and implementation of such QMs can provide the real platform for the construction of universal optical and microwave quantum computers.

### Data availability

The data that support the plots within this paper and other findings of this study are available from the corresponding author upon reasonable request.
